# Goethe and Schiller

**Published:** 1882-06

**Authors:** 


### GOETHE AND SCHILLER.

### THE PERSONAL APPEARANCE OF THE TWO GERMAN POETS.

Goethe had dark brown hair and eyes, the latter large and almost preternaturally luminous. His complexion also was more olive than fair, the nose nearly Roman, but with a Greek breadth at the base, and sensitive, dilating nostrils; the mouth and chin, on the sculptor's line, ample, but so entirely beautiful that they seemed smaller than their actual proportions. His face was always more or less tanned; he rarely lost the brand of the sun. In his later years it became ruddy, and a slight increase of fulness effaced many of the wrinkles of age. Stieler's portrait—now in the Goethe mansion—painted when the poet was eighty, expresses an astonishing vital power. Preller once said to me: "There never was such life in so old a man! If a cannon ball had suddenly grazed my head I could not have been more startled than when I heard of his death. I felt sure that he would live to be 150 years old!" If Goethe illustrates as scarcely any other poet (yet we imagine both Homer and Shakespeare to have possessed the same) the perfect accord of intellectual and physical forces, Schiller is equally remarkable as an example of a mind triumphing over incessant bodily weaknesses and torments. During fourteen years he never knew a day of complete unshaken health. He was fair and freckled, with so delicate a skin that the slightest excitement of his blood blushed through it. His thin, aggravated, aquiline nose was so conspicuous that he often laughingly referred to it as the triumphant result of constant pinching and pulling during his school-days. His chin was almost equally prominent, giving him what his sister Christophine called "a defiant and spiteful under-lip." His shock of hair, not parting into half curls like Goethe's, but straight and long, was of a yellow-brown hue, "shimmering into red," as Caroline von Wolzogan poetically says. The picture of him touches our sympathies, as his bust or statue always does—perhaps because he represents suffering and struggle so palpably. Beside him Goethe seems to stand crowned by effortless achievement. But what a pair they are! Rietschel's great success in his statues lies in his subtle expression of their noble friendship. Goethe's hand on Schiller's shoulder, and the laurel wreath which the hands of both touch, in such wise that you cannot be sure which gives or which takes, symbolize a reality far too rare in the annals of literature.

### THE IDEAL.

I think the song that's sweetest
Is the one that's never sung;
That lies at the heart of the singer
Too grand for mortal tongue.
And sometimes in the silence
Between the day and night,
He fancies that its measures
Bid farewell to the light.

A picture that is fairer
Than all that have a part
Among the masterpieces
In the marble halls of art,
Is the one that haunts the painter
In all his golden dreams,
And to the painter only
A real picture seems.

The noblest, grandest poem
Lies not in blue and gold
Among the treasured volumes
That rosewood bookshelves hold;
But in bright, glowing visions,
It comes to the poet's brain,
And when he tries to grasp it
He finds his effort vain.

A fairy hand from dreamland
Beckons up here and there,
And when we strive to clasp it
It vanishes into air.
And thus our fair ideal
Floats always just before,
And we with longing spirits
Reach for it evermore.

### ASTHMA CURE.

Belladonna leaves, two ounces ; stramonium leaves, one ounce ; powdered nitrate of potassium, one and one-half drachms. Mix them thoroughly. To be used by igniting a drachm in a suitable vessel and inhaling the fumes.

N. B.—A better plan is to go to Texas and remain there.—Ed.

### ROSE SACHET.

Rose leaves, ground, four ounces ; sandalwood, ground, two ounces ; oil of rose, one drachm ; powdered orris root, one ounce. Mix them.

"Two men got into a fight in front of the bank to-day," said a stock-broker at the family supper-table, "and it looked pretty hard for one of them. The biggest one grabbed a cart-stake and drew it back. I felt that he was going to knock the other's brains out, and I jumped between them."

The family had listened with rapt attention, and, as the head paused in his narrative, the young heir, whose respect for his father's bravery was immeasurable, proudly remarked, "He couldn't knock any brains out of you, could he, father?"

The head of the family gazed long and earnestly at the heir, as if to detect evidences of a dawning humorist ; but as the youth continued with great innocence to munch his tart, he gasped and resumed his supper.

### HOW A MAN GOES TO BED.

Speaking of how a man goes to bed, an exchange says :

"There's where a man has the advantage. He can undress in a cold room and have his bed warm before a woman has got her hairpins out and her shoes untied."

That's how it looks in print, and this is how it is in reality : "I am going to bed, my dear. It's half-past ten." No reply. "Now, John, you know you'r always late in the morning. Do get to bed !" "Yes, in a minute," he replies, as he turns the paper wrong side out and begins a lengthy article headed "The Louisiana Muddle." Fifteen minutes later she calls from the bedroom : "John, come to bed, and not keep the gas burning here all night," and murmuring something about "the bill being big enough now," she creeps between the cold sheets, while John sits placidly on, his feet across the piano stool and a cigar in his mouth. By-and-by he rises, yawns, stretches himself, throws the paper on the floor, and seizing the shaker proceeds to that vigorous exercise, shaking the coal stove. Just at this stage a not altogether pleasant voice inquires: "For pity's sake ! ain't you ready for bed yet ?" "Yes, yes, I'm coming ! Why don't you go to sleep and let a fellow alone ?"

Then he discovers that there's coal needed. When that is supplied and rattled into the stove he sits down to warm his feet. Next he slowly begins to undress, and as he stands scratching himself and absently gazing on the last garment, dangling over the back of the chair, he remembers that the clock is not wound yet. When that is attended to he wants a drink of water, and away he promenades to the kitchen. Of course, when he returns his skin resembles that of a picked chicken, and once more he seats himself before the fire for a last "warm-up." As the clock strikes twelve he turns out the gas, and with a flop of the bed-clothes and a few spasmodic shivers he subsides—no, not yet ; he forgot to see if the front door was locked, and another flop of the bed-clothes brings forth the remark : "Good gracious ! if that man ain't enough to try the patience of Job !" Setting her teeth hard she awaits the final flop, with the accompanying blast of cold air, and then quietly inquires "if he is settled for the night," to which he replies by muttering : "If you ain't the provokingest woman !"

